# Hospice Use and Spiritual Support for Family and Friend Caregivers in End of Life and Bereavement

**DOI:** 10.1093/geroni/igaf122.1121

**Published:** 2025-12-31

**Authors:** Allison Kirkegaard, Denise Quigley

**Affiliations:** RAND Corporation, Santa Monica, California, United States; RAND Corporation, Santa Monica, California, United States

## Abstract

As family and friend caregivers navigate the time approaching and following the care recipient’s death, they may benefit from spiritual support. Though access to spiritual support for end of life and bereavement is affected by whether the care recipient receives hospice, which typically includes access to resources such as chaplains and grief support, this connection is underexplored. We use data from 315 recently bereaved caregivers in the 2023 wave of the Care Network Connections over Time (CNCT) study, a nationally representative longitudinal survey of family and friend caregivers helping adults 50 and older, to explore hospice use among care recipients and its relationship to spiritual support among caregivers. Among the 315 caregiving dyads, 60% of care recipients were on hospice at the time of death, with hospice use significantly predicted by rural residence (OR = 0.35, p = 0.04) and non-White and/or Hispanic race/ethnicity (OR = 0.40, p = 0.04). Controlling for sociodemographic and contextual characteristics, caregivers of care recipients on hospice were more likely than caregivers of care recipients not on hospice to both want (OR = 2.66, p = 0.02) and receive (OR = 2.57, p = 0.04) spiritual support before the care recipient’s death. The type of spiritual support received varied by the caregiver’s religious affiliation. During bereavement, there were no significant associations between care recipients’ hospice use and caregivers’ desire for or receipt of spiritual support. These findings suggest that hospice is an important determinant of caregivers’ spiritual support, a key resource for promoting wellbeing, at the end of care recipients’ lives.

